# Dose-dependent effects of dietary quercetin on cecal microbiota, hematological responses, and production efficiency in Arbor Acres broiler chickens

**DOI:** 10.14202/vetworld.2026.264-281

**Published:** 2026-01-25

**Authors:** Shamil Rakhmatullin, Marina Kurilkina, Dianna Kosyan, Dmitry Deryabin, Galimzhan Duskaev

**Affiliations:** Federal Research Center of Biological Systems and Agrotechnologies of the Russian Academy of Sciences, 29, 9 Yanvarya St., Orenburg, 460000, Russia

**Keywords:** antibiotic alternative, broiler chickens, cecal microbiota, feed additive, growth performance, hematological parameters, immune modulation, phytobiotics, poultry nutrition, quercetin, 16S rRNA sequencing

## Abstract

**Background and Aim:**

The gastrointestinal microbiome plays a key role in nutrient absorption, immune regulation, and growth performance in broiler chickens. As restrictions on antibiotic growth promoters increase, phytogenic compounds like quercetin (QC) have gained attention as potential alternatives. Although QC is recognized for its antioxidant and immunomodulatory effects, its dose-dependent influence on gut microbiota composition and systemic immune parameters remains not fully understood. This study aimed to assess the effects of graded dietary QC supplementation on cecal microbiome structure, hematological profiles, and production performance in Arbor Acres (AA) broiler chickens to identify an optimal and safe inclusion level.

**Materials and Methods:**

A total of 180 seven-day-old AA broiler chickens were randomly divided into four groups (n = 45 per group; three replicates). Birds received either a basal diet (BD, control) and the BD supplemented with QC at 5 mg/kg (QC1), 10 mg/kg (QC2), or 15 mg/kg (QC3) of feed daily for 35 days. Growth performance indicators, including body weight gain, feed conversion ratio, livability, and the European Production Efficiency Factor (EPEF), were recorded. Hematological parameters were analyzed using an automated veterinary hematology analyzer. Cecal microbiota composition was examined through high-throughput 16S Ribosomal ribonucleic acid (rRNA) gene sequencing, followed by alpha- and beta-diversity analyses and differential abundance testing.

**Results:**

Dietary QC significantly affected broiler performance, immune status, and gut microbiota composition in a dose-dependent way. The QC1 group achieved the highest final body weight, average daily gain, and EPEF, with an 11.6% increase in production efficiency compared to the control. Hematological analysis showed increased total leukocyte and lymphocyte counts, along with decreased neutrophil, monocyte, eosinophil, and basophil levels, reflecting immunomodulatory and anti-inflammatory effects. Microbiome analysis indicated that Bacillota and Bacteroidota were dominant across all groups. QC at 5 mg/kg boosted beneficial, butyrate-producing genera, especially Faecalibacterium, while preserving microbial balance. Conversely, higher doses (10–15 mg/kg) led to a notable rise in Campylobacterota, suggesting a possible shift toward dysbiosis. Alpha-diversity measures were not significantly affected, but beta-diversity analysis confirmed distinct changes in microbial communities among the treatment groups.

**Conclusion:**

Dietary QC has a clear dose-dependent effect on the gut microbiota–immune–performance axis in broiler chickens. Supplementation at 5 mg/kg of feed is the optimal level, improving growth performance, feed efficiency, immune balance, and beneficial microbial populations without increasing pathogenic taxa. Higher supplementation levels may disturb microbial balance and raise the levels of potentially harmful bacteria. These findings support QC as a promising phytogenic alternative to antibiotic growth promoters and provide a scientific basis for its rational use in sustainable, antibiotic-free poultry production systems.

## INTRODUCTION

Poultry farming is a key part of global animal protein production. The sector’s efficiency depends largely on the birds’ ability to convert feed into muscle, a process heavily influenced by the gastrointestinal microbiota. The gut microbiome is crucial for digesting nutrients, preventing pathogens through competitive exclusion and antimicrobial metabolites, and regulating the host immune system [1]. Therefore, maintaining a balanced, stable gut microbial community is essential for optimal animal health, productivity, and overall welfare [2].

In response to rising legislative restrictions on the use of antibiotic growth promoters due to concerns about antimicrobial resistance, interest in natural feed alternatives has grown [3]. Phytobiotics, including plant-derived flavonoids, have emerged as promising options because they can selectively influence gut microbial communities, improve feed conversion efficiency, and combat pathogenic bacteria such as *Clostridium* and *Escherichia coli* [4–10]. Among these compounds, the flavonol quercetin (QC) has attracted significant attention due to its well-established antioxidant, anti-inflammatory, and immunomodulatory properties in monogastric animals. In broiler chickens, QC supplementation has been shown to improve meat quality, increase nutrient digestibility, and boost both innate and adaptive immune responses [11–15].

Despite increasing evidence supporting the benefits of QC supplementation in poultry diets, significant gaps remain in understanding its dose-dependent biological effects. Most existing studies mainly focus on growth performance, antioxidant levels, or selected immune markers, often evaluating only a single inclusion level or using plant extracts with varying QC purity. As a result, the boundary between beneficial and potentially harmful effects of QC on gut microbial ecology has not been clearly established. Specifically, comprehensive studies linking graded QC supplementation with detailed analysis of the cecal microbiome are limited.

Furthermore, existing studies often focus on overall microbial diversity without analyzing specific changes in important bacterial groups and their functional impact on host immunity and productivity. The relationship between QC-induced changes in the microbiota and systemic immune responses, as reflected in detailed hematological profiles, remains poorly understood. Significantly, the risk that higher doses of QC might disturb microbial balance or promote opportunistic bacteria has received little attention, despite its importance for food safety and sustainable poultry production.

Furthermore, few studies have combined microbiome, hematological, and production efficiency parameters within a single experimental framework. This lack of integration restricts the ability to establish mechanistic links along the gut–microbiota–immune–performance axis and hinders the determination of an optimal, safe, and effective QC inclusion level for broiler diets. Addressing these gaps is crucial for the appropriate use of QC as a phytogenic alternative to antibiotic growth promoters.

The current study was designed to systematically evaluate the dose-dependent effects of dietary QC supplementation on cecal microbiota composition, hematological and immune parameters, and growth performance in AA broiler chickens. Specifically, this study aimed to (i) characterize changes in the structure and composition of the cecal microbiome in response to graded QC inclusion using high-throughput *16S rRNA* gene sequencing; (ii) assess the influence of QC on hematological indicators that reflect immune modulation and inflammatory status; and (iii) determine the impact of different QC doses on key production efficiency metrics, including body weight gain, feed conversion ratio (FCR), livability, and European Production Efficiency Factor (EPEF).

By integrating microbiome, immunophysiological, and performance data into a single experimental model, this study aimed to identify the optimal dietary QC dose that maximizes productive performance while maintaining microbial balance and systemic health. The findings are intended to provide a scientific basis for the safe and effective use of QC as a phytogenic feed additive in antibiotic-free poultry production systems.

## MATERIALS AND METHODS

### Ethical approval

All animal handling, housing, and experimental procedures were carried out in strict accordance with internationally accepted principles for the ethical use of animals in research and relevant national regulations. The study adhered to the provisions of the Model Law of the Interparliamentary Assembly of the Commonwealth of Independent States “On the Treatment of Animals,” Article 20 (Resolution of the IPA of the CIS Member States No. 29-17, October 31, 2007), as well as the Methodological Recommendations for Working with Laboratory Animals.

All experimental protocols involving live animals were reviewed, evaluated, and officially approved by the Animal Care and Use Committee of the Federal Research Center for Biological Systems and Agrotechnologies of the Russian Academy of Sciences in Orenburg, Russia (Approval Protocol No. 4, dated September 30, 2024). The study was designed and conducted in accordance with the principles of Replacement, Reduction, and Refinement (3Rs) to minimize animal use and suffering while maintaining scientific validity.

Broiler chickens were housed in controlled environmental and hygienic conditions suitable for their age and physiological needs, with continuous access to feed and water. Animal health and welfare were checked twice daily by trained personnel under veterinary supervision. No therapeutic drugs, including antibiotics, were given during the experimental period. Criteria for humane intervention and removing animals from the experiment were predetermined and included severe injury, losing more than 15% of body weight, inability to access feed or water on their own, or signs of incurable disease.

Blood sampling and tissue collection were performed by trained personnel using standard veterinary procedures to minimize stress and discomfort. At the end of the experiment, birds selected for hematological and microbiome analyses were humanely euthanized with an intravenous injection of pentobarbital (150 mg/kg body weight) via the wing vein, in line with accepted veterinary guidelines. Death was confirmed before necropsy and sample collection.

Throughout the study, all efforts were made to ensure animal welfare, reduce distress, and adhere to ethical standards for animal research.

### Study duration and location

The experiment was conducted from November 2024 to July 2025 at the Experimental Biological Clinic (vivarium) of the Federal Research Center for Biological Systems and Agrotechnologies of the Russian Academy of Sciences in Orenburg, Russia (51.7727° N, 55.0988° E). The trial was performed under fully controlled, climate-regulated housing conditions to maintain consistent environmental parameters throughout the entire experimental period.

### Experimental animals and study design

A total of 180 AA broiler chickens (supplier: LLC “Aviagen,” Tula, Russia), aged 7 days at the start of the experiment, were included in the study. Birds were grouped by live body weight using 10-g intervals and randomly assigned to four experimental groups (n = 45 per group) to ensure similar initial weights. Each group was then divided into three replicates of 15 birds each. All chicks were individually marked with colored plastic leg rings.

The average initial body weight on day 7 was 203.8 ± 1.5 g (mean ± Standard error of the mean [SEM]). Birds were of mixed sex, and stratified randomization ensured a balanced sex ratio across all groups. The experiment lasted 42 days, including a 7-day acclimation period followed by a 35-day experimental phase. The experimental layout is shown in Table 1.

**Table 1:** Experimental design showing grouping, dietary treatments, and duration of the preparatory and experimental periods in Arbor Acres broiler chickens.

Object of the study	Group	Experiment period	

		Preparatory (7 days)	Accounting period (35 days)
Broiler chickens "Arbor Acres"	Control (n = 45)	BD	BD
	Experiment I (n = 45)	BD	QC1
	Experiment II (n = 45)	BD	QC2
	Experiment III (n = 45)	BD	QC3

BD = Basal diet, QC = Quercetin, QC1 = BD supplemented with quercetin at 5 mg/kg of feed/day, QC2 = BD supplemented with quercetin at 10 mg/kg of feed/day, QC3 = BD supplemented with quercetin at 15 mg/kg of feed/day.

At the end of the experiment, nine birds from each group were randomly chosen for hematological and microbiome analyses. Sampling was proportional, with one bird selected from each cage, resulting in three birds per replicate and nine per treatment group.

### Housing and environmental management

Broilers were housed in three-tier cages measuring 0.9 × 0.45 m, with five birds per cage, resulting in a stocking density of 0.08 m² per bird. The housing facility had a mechanical ventilation system to ensure continuous air exchange. Ambient temperature was initially maintained at 32°C and gradually decreased by 0.5°C daily until reaching 20°C–22°C. Relative humidity was kept between 50% and 70%.

A controlled lighting program was used with a light intensity of 20–25 lux at bird level: 23 h light and 1 h darkness from days 1–7, 20L:4D from days 8–21, and 18L:6D from days 22–42. This lighting schedule complied with European Council Directive 2007/43/EC on the protection of broiler chickens. Strict sanitary measures, including regular cleaning and disinfection of cages and equipment, were maintained throughout the study.

### Diet formulation and QC supplementation

Birds were fed and watered on a group basis. QC dihydrate (purity ≥ 95%; 95+% AL33795-1, Sigma-Aldrich, China) was used as the dietary supplement and stored according to the manufacturer’s instructions. The control group received a basal diet (BD) formulated to meet nutritional requirements (Table 2).

**Table 2:** Nutritional composition of the basal diet provided to broiler chickens during the starter and finisher phases.

Indicator	Starting composition	Growth composition
Moisture	10.67	8.50
Dry matter	89.33	91.50
Crude protein	23.20	22.10
Crude fat	4.80	4.81
Crude fiber	5.90	4.40
Ash	5.80	6.20

Values are expressed on an as-fed basis and presented as g/kg.

Experimental diets were supplemented with QC dihydrate as follows: group QC1 received BD + 5 mg/kg feed/day, group QC2 received BD + 10 mg/kg feed/day, and group QC3 received BD + 15 mg/kg feed/day. QC was first mixed thoroughly with a small amount of BD to create a uniform premix, which was then incorporated into the total feed using a horizontal mixer for 15 minutes to ensure even distribution. Nutrient and trace element contents of the diets met broiler requirements for each growth phase (Table 3) [16].

**Table 3:** Ingredient composition and calculated nutrient content of the basal diet used during the starter and finisher phases of broiler production.

Attributes	Starter (7–28 days)	Finisher (29–42 days)
	
	Control, I, II, and III	Control, I, II, and III
Ingredient composition (%)		
Wheat	48.0	44.0
Barley	3.2	0.9
Corn	10.0	21.0
Soybean meal (46% CP)	23.0	17.0
Sunflower meal (38% CP)	5.0	10.0
Sunflower oil	5.0	5.0
Dicalcium phosphate	1.6	1.6
Mel stern	0.9	1.5
Limestone	0.5	0.3
Salt	0.32	0.22
DL-Methionine	0.18	0.16
L-Lysine	0.35	0.17
Vitamin-mineral premix*	2.0	2.0
Calculated nutrients Metabolizable energy (kkal/100 g)	298.0	305.0
CP	24.0	19.0
Methionine + cysteine	0.87	0.79
Lysine	1.35	0.96
Calcium	0.95	1.0
Available phosphorus	0.54	0.48

CP = Crude protein.

* Vitamin-mineral permit (VetAgro, Russia) per kilogram of diet: Vitamin A: 7.000 IU, Vitamin D3: 800.0 IU, Vitamin E: 9 IU, Vitamin K3: 1.1 mg, Thiamin: 1.0 mg, Riboflavin: 5.0 mg, Vitamin B6: 2 mg, Vitamin B12: 0.05 mg, Vitamin C: 70 mg, Mn: 25 mg, Fe: 15 mg, Zn: 11 mg, Cu: 2.5 mg, I: 0.4 mg, Se: 0.5 mg, CP = Crude protein.

### Health monitoring and welfare management

All birds followed a standard commercial vaccination program for AA broilers in the Russian Federation. Vaccination started at the hatchery with Marek’s disease vaccine (CV1988/Rispens strain) applied subcutaneously, along with a combined infectious bronchitis and Newcastle disease vaccine delivered by coarse spray. Later vaccinations included infectious bronchitis vaccine (Ma5 strain) on day 5, infectious bursal disease vaccine (Winterfield R strain) on day 10, and booster shots against Newcastle disease (La Sota strain) and infectious bronchitis (Ma5 strain) on day 16 via drinking water.

Vaccines were administered using approved commercial products under veterinary supervision. No therapeutic drugs, including antibiotics, were used during the experiment. Bird health was monitored twice daily for behavior, general condition, and signs of distress. Mortality and euthanasia events were recorded daily with documented causes. Criteria for euthanasia included loss of more than 15% body weight, inability to access feed or water, severe injury, or incurable infection.

### Blood collection and hematological analysis

At the end of the experiment, blood samples were collected from the wing vein of nine randomly selected birds per group into 2-mL EDTA tubes. Sampling was performed in the morning before feeding to reduce diurnal variation. Each sample was tested in triplicate.

Following blood collection, birds were immediately euthanized with an intravenous dose of pentobarbital (150 mg/kg body weight). Hematological parameters, including hemoglobin, hematocrit, red blood cell count, white blood cell count, and leukocyte differential, were measured using an automated hematology analyzer (URIT-2900 Vet Plus; URIT Medical Electronic Co., China). The analyzer was calibrated beforehand, and quality control was maintained with commercial avian blood control materials.

### Cecal sample collection and storage

Immediately after euthanasia on day 42, cecal contents were collected aseptically from the same birds used for hematological analysis. Sterile instruments were employed for each bird to prevent cross-contamination. About 200 µL of cecal content was transferred into sterile cryogenic tubes containing 200 µL of DNA/RNA Shield preservative (Zymo Research, USA). Samples were promptly frozen on dry ice and stored at −80°C until DNA extraction. The time between slaughter and sample freezing did not exceed 10–15 minutes.

### Growth performance evaluation

Individual live body weights were recorded weekly. Feed intake was measured daily per cage by recording the feed offered and residual feed. Average daily gain (ADG), FCR, and EPEF were calculated using standard formulas. Higher EPEF values indicated better overall production efficiency.

### DNA extraction and 16S rRNA sequencing

Total genomic DNA was extracted from cecal samples using the FastDNA SPIN Kit for Feces (MP Biomedicals, USA). DNA concentration and purity were determined using a Qubit 4 fluorometer and confirmed by agarose gel electrophoresis.

The V3–V4 region of the *16S rRNA* gene was amplified using primers 341F and 785R following Illumina’s standard protocol. Libraries were purified with AMPure XP beads and sequenced with paired-end 2 × 250 bp chemistry on an Illumina MiSeq platform.

### Bioinformatic processing and taxonomic classification

Raw sequencing data were processed with the UPARSE pipeline (USEARCH v11) [17] After quality filtering and Operational taxonomic unit (OTU) clustering at 97% similarity, taxonomic classification was carried out using the Ribosomal Database Project Classifier against the SILVA v138 database. Taxonomy was further refined using the GTDB and National Center for Biotechnology Information databases. Downstream analyses were performed with MicrobiomeAnalyst 2.0 [18].

### Data filtering, normalization, and diversity analysis

Low-abundance and low-variance OTUs were removed before analysis [19]. Data normalization was done using cumulative sum scaling and total sum scaling. Samples were rarefied to 20,000 reads per sample for diversity analysis. Alpha-diversity indices (Chao1, Shannon, Simpson) and beta-diversity metrics (Bray–Curtis, Jensen–Shannon) were calculated and visualized through Principal coordinate analysis and Non-metric multidimensional scaling ordinations.

### Statistical analysis

All data, including performance parameters, hematological indicators, and alpha-diversity indices, were initially assessed for normality of distribution using the Shapiro–Wilk test and for homogeneity of variances using Levene’s test. Data that satisfied the assumptions of normality and homoscedasticity were analyzed with one-way analysis of variance (ANOVA), followed by Tukey’s post hoc test for multiple comparisons. Data that did not meet these assumptions were analyzed using nonparametric statistical methods, specifically the Kruskal–Wallis test followed by Dunn’s post hoc test with Benjamini–Hochberg correction.

All results are shown as mean ± SEM. Statistically significant differences between experimental groups at p ≤ 0.05 are indicated by different lowercase letters within the same row of the tables.

To assess differences in the overall structure of microbial communities (beta-diversity), nonparametric multivariate ANOVA was conducted using the Bray–Curtis similarity measure with 999 permutations. Differential abundance of microbiome taxa between experimental groups was analyzed with the EdgeR package. The resulting p-values were adjusted for multiple comparisons using the false discovery rate correction.

For subsequent pairwise comparisons of relative taxon abundance, either parametric methods (ANOVA followed by Tukey’s test) or nonparametric methods (Mann–Whitney U-test) were used, depending on whether the data met the assumptions required for each test. All statistical comparisons and graphical visualizations, including bar charts of taxonomic composition, ordination plots, and core microbiome analyses at the phylum and genus levels, were carried out using MicrobiomeAnalyst 2.0 and PAST software [20].

Statistical analyses were conducted using SPSS version 26.0 (IBM Corp., NY, USA) and Statistica version 20.0 (TIBCO Software Inc., USA). Raw data and derived variables were managed with Microsoft Excel 2023 (Microsoft Corporation, USA).

## RESULTS

### Growth performance and feed conversion efficiency

Analysis of live weight dynamics (Table 4) showed that all experimental groups outperformed the control group in terms of growth. The highest weight gain was observed in the QC1 group (5 mg/kg). On day 42, the birds reached an average weight of 2610 g (p ≤ 0.05), which was 10.1% higher than the control group (2371 g). The ADG was also highest in the QC1 group (68.74 g/day vs. 61.86 g/day in the control group). Groups QC2 and QC3 displayed intermediate values, indicating a dose-dependent effect of QC, with optimal results at the lowest dose.

**Table 4:** Body weight of broiler chickens receiving different dietary levels of quercetin during the experimental period (g, mean ± SEM).

Experiment duration (days)	Group			

	Group I (QC1)	Group II (QC2)	Group III (QC3)	Control (BD)
7	204.00 ± 11.9	203.71 ± 10.7	203.86 ± 10.4	203.86 ± 10.7
14	464.43 ± 27.4	411.57 ± 23.5	433.43 ± 20.6	429.43 ± 19.6
21	954.00 ± 55.9	851.14 ± 40.5	882.86 ± 38.9	821.57 ± 33.7
28	1423.57 ± 76.2	1293.14 ± 59.0	1324.57 ± 54.6	1212.86 ± 63.7
35	1921.14 ± 87.2^a^	1806.50 ± 95.8	1812.33 ± 80.4	1633.00 ± 97.3
42	2610.00 ± 115.7^a^	2443.83 ± 125.3	2528.17 ± 89.4	2371.20 ± 119.3
Average daily growth over the entire experiment	68.74 ± 3.1	64.08 ± 3.7	66.33 ± 2.3	61.86 ± 3.0

SEM = Standard error of the mean. Mean values within the same row with different superscript letters differ significantly compared with the control group (p ≤ 0.05).

The evaluation of feed intake and its conversion (Table 5) confirmed the benefits of the QC1 group: despite having the highest total feed intake (4201.89 g), this group achieved the best FCR (1.77 g feed per kg of gain vs. 1.80 g in the control). Livability in groups QC1 and QC3 was 98%, compared to 95% in the control, indicating a positive effect of QC on bird survival.

**Table 5:** Feed intake, feed conversion ratio, and livability of broiler chickens fed diets supplemented with different levels of quercetin.

Parameter	Group			

	Group I (QC1)	Group II (QC2)	Group III (QC3)	Control (BD)
Consumption for the entire experiment (g)	4201.89	4000.45	4049.14	3862.09
FCR (g)	1.77	1.81	1.75	1.80
Livability (%)	98	97	98	95

**Figure 1:**
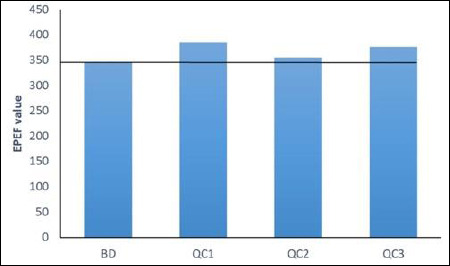
EPEF of broiler chickens fed diets supplemented with graded levels of QC during the experimental period. EPEF = European Production Efficiency Factor, BD = Basal diet, QC = Quercetin, QC1 = Quercetin at 5 mg/kg of feed, QC2 = Quercetin at 10 mg/kg of feed, QC3 = Quercetin at 15 mg/kg of feed.

The standardized EPEF was used for a comprehensive comparison of performance results (Figure 1). The EPEF values in experimental groups I and III were 386.2 and 375.9 units, respectively, compared to 346 in the control, which was 40.2 and 29.9 units higher; in experimental group II, the difference from the control value was 9.5 units.

### Hematological and immune parameters

Blood analysis (Table 6) revealed that QC had a significant effect on immunological and physiological parameters. Group QC2 (10 mg/kg) showed the highest levels of leukocytes (48.11 × 10^9^/L) and lymphocytes (58.7%), indicating immune system stimulation. In group QC1, an increase in hemoglobin level was observed (121.25 g/L vs. 107.67 g/L in the control), likely linked to improved oxygen transport. Meanwhile, the 15 mg/kg dose (QC3) did not show additional benefits and, for some parameters (neutrophils and monocytes), approached control values.

**Table 6:** Hematological parameters of broiler chickens fed diets supplemented with graded levels of quercetin (Mean ± SEM).

Parameter	Group			

	Control (BD)	Group I (QC1)	Group II (QC2)	Group III (QC3)
WBC, 10^9^ cells/L	36.03 ± 2.36	42.29 ± 1.20^a^	48.11 ± 1.24^a^^b^	40.19 ± 1.25^c^
LYM, %	52.43 ± 0.43	55.38 ± 2.08	58.70 ± 1.84^a^	57.95 ± 1.38^a^
MON, %	0.47 ± 0.03	0.33 ± 0.02^a^	0.23 ± 0.05^a^	0.48 ± 0.13
RBC, 10^12^ cells/L	2.17 ± 0.21	2.24 ± 0.17	2.21 ± 0.05	2.12 ± 0.04
HGB, g/L	107.67 ± 3.18	121.25 ± 8.67	117.75 ± 1.80^a^	112.50 ± 2.47
HCT, %	24.33 ± 0.75	28.43 ± 2.22	25.88 ± 0.46	25.30 ± 0.53
NEU, %	44.33 ± 0.43	35.65 ± 2.15^a^	38.35 ± 2.32^a^	45.78 ± 2.66^b^^c^
EOS, %	11.60 ± 1.08	4.43 ± 0.15^a^	7.78 ± 0.49^a^^b^	7.70 ± 0.44^a^^b^
BAS, %	0.67 ± 0.03	0.80 ± 0.04^a^	0.45 ± 0.06^a^^b^	0.43 ± 0.08^a^^b^
NEU, 10^9^ Cells/L	15.33 ± 0.93	15.50 ± 0.87	18.93 ± 1.36^b^	18.76 ± 0.78^a^^b^
LYM, 10^9^ Cells/L	24.53 ± 0.90	18.73 ± 0.76^a^	25.45 ± 1.32^b^	26.35 ± 0.94^b^
PLT, 10^9^ Cells/L	1.33 ± 0.33	1.50 ± 0.29	2.25 ± 0.48	2.25 ± 0.25
MON, 10^9^ Cells/L	0.44 ± 0.07	0.22 ± 0.01^a^	0.13 ± 0.01^a^^b^	0.18 ± 0.01^a^^b^
EOS, 10^9^ Cells/L	4.13 ± 0.10	6.03 ± 0.18^a^	3.63 ± 0.14^a^^b^	3.16 ± 0.24^a^^b^
BAS, 10^9^ Cells/L	0.48 ± 0.04	0.58 ± 0.03^a^	0.20 ± 0.02^a^^b^	0.17 ± 0.03^a^^b^

WBC = White blood cells, RBC = Red blood cells, HGB = Hemoglobin, HCT = Hematocrit, LYM = Lymphocytes, MON = Monocytes, NEU = Neutrophils, SEM = Standard error of the mean. Mean values within the same row with different superscript letters differ significantly (p ≤ 0.05).

### Cecal microbial composition at the phylum level

Comparative analysis of the microbial community in the cecal contents of birds from the control and experimental groups revealed several significant changes caused by introducing small plant-derived molecules, such as QC at doses of 5, 10, and 15 mg/kg, which have been previously shown to inhibit bacterial communication systems reliant on population density. At the highest taxonomic level, two phyla were dominant across all groups: *Bacillota* (ranging from 51.12% in the control group to 73.88% in group I) and *Bacteroidota* (from 22.63% in group I to 42.01% in the control group).

The introduction of QC at various concentrations into the BD caused a significant (2–3 fold) increase in the proportion of representatives of the phylum *Campylobacterota* in groups II and III compared to the control group, along with a sevenfold increase in intra-group comparison relative to group I. This finding warrants attention, as this phylum includes many human, animal, and bird pathogens. Therefore, adding high concentrations of QC to the diet promotes an increase in pathogenic agents, with a clear dose-dependent relationship: the higher the concentration, the greater their proportion.

In group I, there was a twofold increase in the number of representatives of the phylum *Actinomycetota* and a twofold decrease in the number of *Candidatus Melainabacteria* representatives compared to groups II, III, and the control. Changes in the balance of other phyla were not significantly noticeable; specifically, there were no significant differences in the relative abundance of representatives of the phyla *Pseudomonadota*, *Thermodesulfobacteriota*, and *Verrucomicrobia* either within groups or when compared to the control (Table 7).

**Table 7:** Relative abundance of dominant bacterial phyla in the cecal microbiota of broiler chickens fed diets supplemented with different levels of QC.

Phylum	Group			

	Group I (QC1)	Group II (QC2)	Group III (QC3)	Control (BD)
*Actinomycetota*	0.21	0.08	0.12	0.10
*Bacillota*	73.88	60.20	55.11	51.12
*Bacteroidota*	22.63	31.00	33.71	42.01
*Campylobacterota*	0.00	4.51	7.05	2.36
*Candidatus_Melainabacteria,*	0.75	1.70	1.50	1.49
*Pseudomonadota*	2.26	2.10	2.05	2.16
*Thermodesulfobacteriota*	0.18	0.20	0.22	0.29
*Verrucomicrobia*	0.01	0.00	0.04	0.02
Unclassified bacteria	0.07	0.20	0.21	0.46

Values are expressed as relative abundance percentages. QC = Quercetin, QC1 = Quercetin at 5 mg/kg of feed, QC2 = Quercetin at 10 mg/kg of feed, QC3 = Quercetin at 15 mg/kg of feed.

### Shifts in cecal microbiota at the genus level

**Figure 2:**
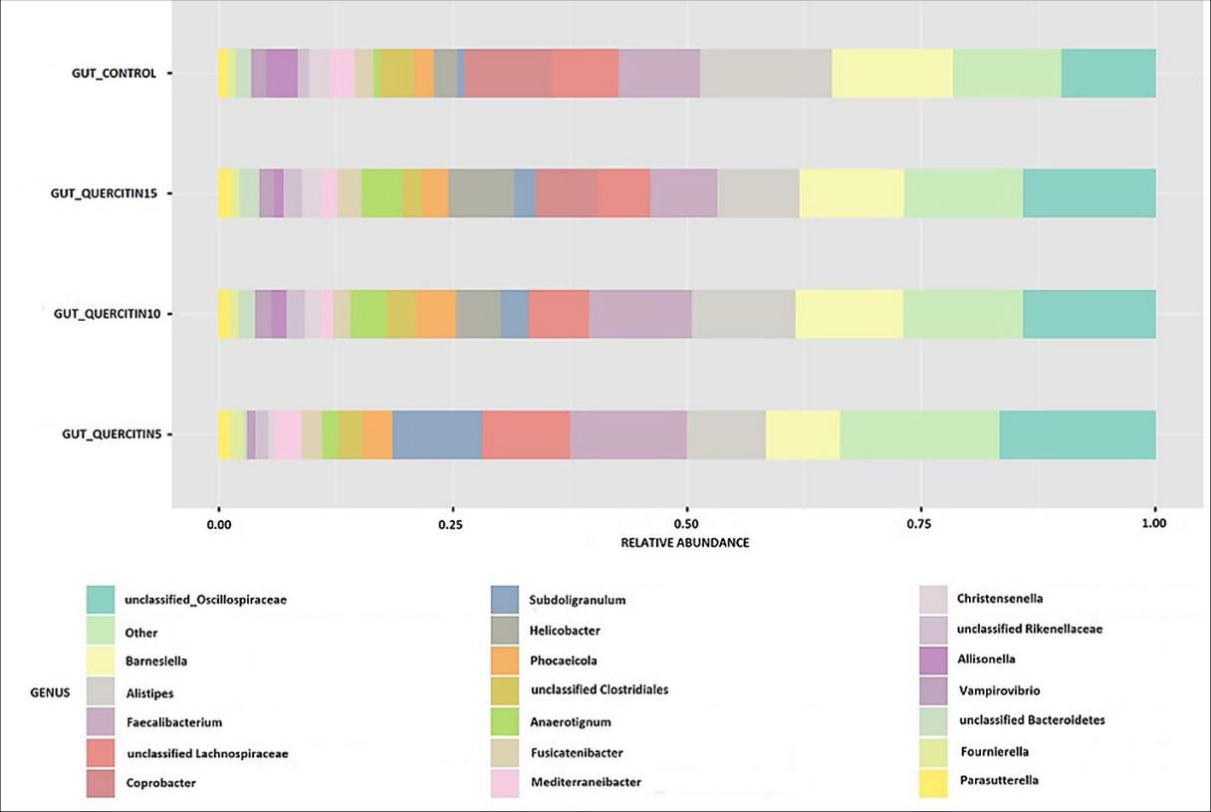
Relative abundance of dominant bacterial genera in the cecal microbiota of broiler chickens receiving different dietary levels of QC. The histogram shows the 20 most represented by the number of births. QC = Quercetin, QC1 = Quercetin at 5 mg/kg of feed, QC2 = Quercetin at 10 mg/kg of feed, QC3 = Quercetin at 15 mg/kg of feed.

Analysis of lower taxonomic ranks (Figure 2) revealed 20 main genera that dominated in relative abundance. In the control group, the primary representatives were *Alistipes* (14%), *Barnesiella* (12%), *Coprobacter* (9%), and *Faecalibacterium* (9%). Among the unclassified genera, most were unclassified *Oscillospiraceae* (10%) and unclassified *Lachnospiraceae* (7%). The abundance of other genera ranged from 4% to 1%. Genera with less than 1% abundance were grouped into the “Other” category, which accounted for 11% of the overall microbiome diversity.

Adding QC at various concentrations caused changes in the microbiome at the genus level. In the first experimental group, the dominant genera were *Faecalibacterium* (12%), *Subdoligranulum* (10%), *Alistipes* (8%), and *Barnesiella* (8%). Among the unclassified types, unclassified *Oscillospiraceae* and unclassified *Lachnospiraceae* stood out, with abundances of 17% and 9%, respectively. The proportions of other genera ranged from 3% to 1%. The “Other” category accounted for 13%.

For experimental group II, the dominant genera were *Barnesiella* (12%), *Alistipes*, and *Faecalibacterium* (11% each); among unclassified forms, unclassified *Oscillospiraceae* and unclassified *Lachnospiraceae* were also noted at 14% and 6%, respectively. The “Other” category accounted for 12%.

In experimental group III, representatives of the genera *Barnesiella* (11%), *Alistipes*, and *Faecalibacterium* (9% and 7%, respectively) also dominated, but genera such as *Helicobacter* and *Coprobacter* (7% each) were also observed. The unclassified genera *Oscillospiraceae* and *Lachnospiraceae* made up 14% and 6%, respectively. The “Other” category accounted for a quarter of the total microorganisms in the cecal microbiome of broilers.

When comparing the experimental and control groups, several clear changes were observed. With the addition of QC, the number of members of the genus *Faecalibacterium* increased by 1.2 and 1.4 times in experimental groups I and II, respectively; however, the most noticeable change was seen with the genus *Subdoligranulum*, whose abundance in experimental groups I, II, and III increased by 14, 4, and 3 times, respectively, compared to the control. An inverse relationship between this effect and QC concentration was noted.

Representatives of this genus can have both positive and negative effects on the host as their numbers increase [21, 22]. If an increase in *Subdoligranulum* is linked with a rise in the genus *Gemmiger*, it may harm the development of experimental birds, since recent studies show a negative correlation between *Subdoligranulum* abundance and body weight. However, if an increase in *Subdoligranulum* occurs alongside *Faecalibacterium* [23, 24], a positive trend in body weight indicators is seen. Additionally, increasing QC concentration to 10 and 15 mg/kg also led to a 1.5-fold increase in representatives of the genus *Helicobacter*, compared to groups II and III; these bacteria were not detected in the control group or group I.

### Alpha and beta-diversity of the cecal microbiome

**Figure 3:**
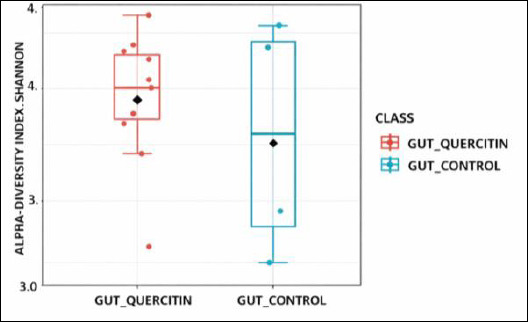
Alpha-diversity of the cecal microbiota in broiler chickens fed diets supplemented with graded levels of quercetin, expressed using the Shannon diversity index.

**Figure 4:**
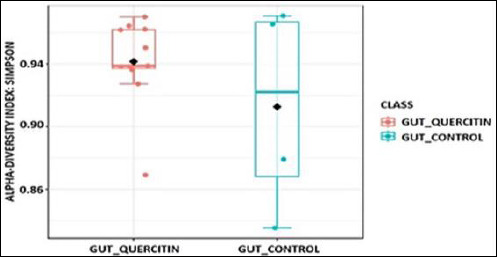
Alpha-diversity of the cecal microbiota in broiler chickens receiving different levels of dietary quercetin, expressed using the Simpson diversity index.

Assessment of alpha-diversity indices revealed that adding QC at different doses did not significantly impact species richness or evenness in the cecal microbial community (Table 8). The Chao-1 index, which measures expected species richness, was highest in group I (QC1, 41.6) receiving 5 mg/kg QC, although its values were similar to those of the control group across other experimental groups. The Shannon (Shannon_H) and Simpson (Simpson_1-D) indices, which consider both richness and evenness, also showed no significant differences between groups (Figures 3 and 4).

**Table 8:** Alpha-diversity indices of the cecal microbiota in broiler chickens receiving graded dietary quercetin supplementation.

Diversity indexes	Group			

	Control (BD)	Group I (QC1)	Group II (QC2)	Group III (QC3)
Chao-1	32.5	41.6	32.5	30,00
Shannon_H	2.798	2.748	2.775	2.872
Dominance_D	0.07966	0.08828	0.08507	0.07491
Simpson_1-D	0.9203	0.9117	0.9149	0.9251

Chao1 = Species richness estimator, Shannon = Shannon diversity index, Simpson = Simpson diversity index. No statistically significant differences were observed among groups (p > 0.05). QC = Quercetin, QC1 = Quercetin at 5 mg/kg of feed, QC2 = Quercetin at 10 mg/kg of feed, QC3 = Quercetin at 15 mg/kg of feed.

**Figure 5:**
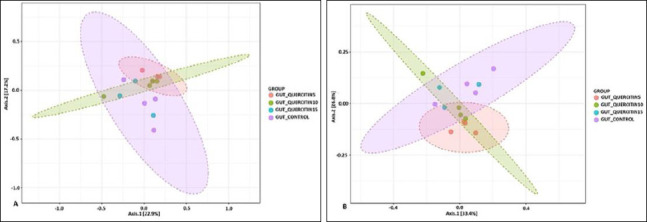
Beta-diversity analysis of the cecal microbiota of broiler chickens based on (A) Bray–Curtis and (B) Jensen–Shannon dissimilarity indices, showing clustering of microbial communities among dietary treatment groups.

In contrast, beta-diversity analysis showed structural differences in microbial community composition between the control and experimental groups, as illustrated with non-metric multidimensional scaling plots based on Bray–Curtis and Jensen–Shannon indices (Figure 5). The clear separation of clusters suggests that QC mainly affected microbial composition rather than overall diversity.

## DISCUSSION

### Overall interpretation of the findings

The study results show a clear dose-dependent effect of QC on broiler performance, health, and cecal microflora. This research offers a comprehensive analysis that combines three related aspects: growth metrics, immunophysiological status (based on hematology data), and gut microbiota composition. Studying these components together is innovative in phytobiotic research.

### Influence of phytobiotics and QC specificity

The gut microbiome is essential for gut health, immunity, and broiler performance [25]. Feed quality greatly influences gut health [26], while phytogenic additives enhance gut microbiota, increasing its efficiency [27]. However, how antimicrobial growth promoters boost bird growth remains unclear; it is believed they enhance growth by modulating the gut microbiota [28]. A healthy gut with a balanced microbial population is vital for improving feed conversion, defending against pathogens, and increasing bird growth rates [29]. Beneficial microbes, such as *Lactobacillus*, are critical for gut functions like metabolism, energy transfer, and growth.

Conversely, an increase in harmful pathogens (e.g., *E. coli*, *Salmonella*, and *Clostridium*) can significantly disrupt the balanced gut ecosystem and hinder bird growth [30]. Phytogenic compounds lower the growth of pathogenic microorganisms [31] and enhance microbiota balance and gut function [32]. Therefore, phytogenic additives can boost bird health and performance [33]. Phytobiotics have antimicrobial properties due to their polyphenolic compounds, including flavonoids like QC. Antimicrobial activity is considered a key feature of phytochemicals used as growth promoters in poultry diets [34]. QC is widely recognized for its antibacterial and antioxidant properties, as well as its ability to improve gut health, stimulate growth, and modulate immunity [35].

### Quorum sensing (QS) interference as a potential mechanism

In addition to the direct antimicrobial and antioxidant properties, our results highlight another potential mechanism: interference with bacterial QS. The dose-dependent restructuring of the microbiota, especially the suppression of certain taxa and the promotion of others, aligns with the action of a QS inhibitor. Previously, *in vitro* experiments have shown that QC antagonizes QS systems in pathogens like *Pseudomonas aeruginosa*, inactivating regulatory molecules and reducing biofilm formation [36].

Our study offers initial evidence that QC’s known properties as a QS inhibitor might also influence the regulation of commensal microbial networks, thereby maintaining leukocyte inflammatory responses. We propose that QC could act similarly within the ceca of broilers, disrupting cell-to-cell communication required for the production of virulence factors and enabling colonization by potential pathogens. This QS-inhibiting activity might explain the decrease in potential pathobionts without completely eliminating all bacteria, thus preserving overall microbial diversity and supporting a more stable gut ecosystem. This method of interfering with bacterial signaling presents a complex mode of action that sets phytobiotics apart from traditional antibiotics and lessens the selective pressure for resistance development [37, 38]

### Performance responses and dose-dependent effects of QC

The results of this study show a clear effect of QC on the performance indicators of AA broiler chicks. The data obtained align well with modern understanding of flavonoid action mechanisms in poultry. The most notable increase in live weight was seen in the group receiving 5 mg/kg of QC (QC1). By day 42 of the experiment, the difference between the control and experimental groups was 238.8 g (2610.00 vs. 2371.20 g), representing a 10.1% increase.

Our results, showing significant growth stimulation at 5 mg/kg, align with data from Sohaib M. *et al*. [39], who reported an 8%–12% increase in final live weight. However, it is very important to note that the source and purity of QC can greatly influence its bioavailability and effectiveness. For example, Zhang S. and Kim I. H. [40] used plant-derived QC at higher doses (0.25–1.00 g/kg) to achieve similar benefits.

This discrepancy might be due to differences in chemical form (aglycone versus glycoside) and to the presence of co-extracts in plant preparations, which could improve stability or absorption. In contrast, our study used highly purified (>95%) QC dihydrate, which could explain the significantly lower effective dose. Variations in BD composition, broiler genetics (AA versus Ross), and health status across studies create different baseline conditions upon which phytobiotics act, making direct dose comparisons challenging. Abid *et al*. [41] found that feeding broilers diets containing 0.1, 0.2, or 0.3 g/kg QC extracted from acacia improved feed conversion.

### Mechanisms underlying growth stimulation

The growth-promoting effect of QC in poultry seems to involve several factors. First, as shown by El-Kazaz *et al*. [42], QC demonstrates strong antioxidant activity, lowering tissue malondialdehyde levels by 25%–30% and boosting superoxide dismutase activity by 15%–20%. This helps create ideal conditions for cellular metabolism and protein production.

Second, according to Abdel-Latif *et al*. [43], QC increases the height and depth of the small intestinal villi, significantly expanding the absorptive surface area of the intestine. Therefore, flavonoids have a beneficial effect on the digestive system of chicks, restoring microbiota balance and enhancing nutrient absorption [44].

### Link between microbiome modulation and performance

The observed rise in the genus *Faecalibacterium*, especially in the QC1 group, is highly noteworthy. *Faecalibacterium prausnitzii*, a key species in this genus, is a primary producer of the short-chain fatty acid butyrate. Butyrate not only provides a major energy source for colonocytes but also acts as a potent immunomodulator. It encourages the differentiation of regulatory T cells and suppresses the production of pro-inflammatory cytokines such as IL-6 and TNF-α [45].

This anti-inflammatory mechanism offers a plausible explanation for the observed hematological changes, including decreased monocyte and neutrophil levels, and supports the improved vitality and overall health status in the QC-supplemented groups. Therefore, the increase in *Faecalibacterium* likely resulted in higher butyrate production, which reduced inflammation levels and enhanced feed conversion, ultimately reflecting improved growth performance.

### Phytobiotic optimum and microbial dysbiosis at higher doses

The clear dose-dependent effect of QC is a notable aspect of the results. Unlike previous reports that used a single-dose, this study shows a nonlinear, biphasic response: moderate inclusion promotes eubiosis, while higher levels disturb microbial balance. This dose-dependent opposition introduces a new idea of a “phytobiotic optimum” in broiler nutrition, identifying 5 mg/kg QC as a beneficial threshold beyond which microbial dysbiosis may happen.

Unlike previous single-dose reports, this study describes a nonlinear, biphasic effect of QC, where moderate inclusion supports eubiosis, while higher levels disrupt microbial balance. This phenomenon is well documented in previous studies [46–51], which show that high doses of flavonoids can have a pro-oxidant effect and inhibit certain metabolic pathways.

The dose-dependent increase in the phylum *Campylobacterota* at 10 and 15 mg/kg is a key point for application. A potential mechanistic hypothesis is suggested for this phenomenon. QC may exert selective pressure at higher concentrations, promoting more resistant bacteria. While beneficial bacteria like *Faecalibacterium* may thrive at lower doses, higher doses could induce a pro-oxidant effect or cause a redox imbalance in the gut environment that some pathogens tolerate better. Additionally, the strong antibacterial activity of QC against dominant beneficial bacteria may reduce competitive exclusion, unintentionally creating a niche for the growth of opportunistic members of the *Campylobacterota* phylum. This highlights the importance of finding the optimal, rather than the maximum, phytobiotic supplement dosage.

### Growth dynamics, feed utilization, and livability

The pattern of live weight changes during the rearing periods should be carefully observed. In the first 14 days of the experiment, differences between groups were minimal. However, starting from day 21, the QC1 group began to show a clear advantage. This aligns with the idea that QC’s effects are most noticeable during the intensive growth phase, when the body’s antioxidant defenses are under the greatest stress [52–54].

The analysis of feeding parameters revealed several key patterns. The total feed intake during the experimental period in the QC1 group was 4201.89 g, which was 8.8% higher than that in the control group (3862.09 g). Despite the higher intake, the FCR in this group was lower (1.77 vs. 1.80 in the control group). These findings suggest that QC not only stimulates appetite but also improves nutrient metabolic utilization. Similar results were reported by Fathima *et al*. [55], who attributed this effect to increased activity of digestive enzymes (amylase by 15%–20%, lipase by 10%–12%) under the influence of flavonoids.

Notably, all experimental groups maintained high livability (97%–98% vs. 95% in the control group). This metric is especially important in industrial poultry farming, as even a slight decrease in mortality can yield significant economic gains in large-scale production. According to data from several researchers, this effect of QC is linked to its ability to decrease the incidence of sudden death syndrome by 30%–35% and gastrointestinal disorders by 20%–25% [56, 57].

### Impact on immune status

Hematological data provide important insights into QC. The most notable changes were seen in WBC parameters. Groups QC1 and QC2 showed significant (p ≤ 0.05) increases in total WBC count of 17.4% and 33.5%, respectively, compared to the control. These findings align with existing data [58], showing QC’s ability to promote hematopoiesis in the red bone marrow through activation of the transcription factor NF-κB.

The dynamics of lymphocyte-mediated immunity are especially noteworthy. Groups QC2 and QC3 showed a significant increase in relative lymphocyte count, with the most notable effect observed in QC3 (26.35 × 10^9^ cells/L vs. 24.53 × 10^9^ cells/L in the control). These changes suggest activation of specific immunity, as reported by Phillips *et al*. [59], who observed a 25%–30% increase in antibody titers against vaccine strains when QC was used.

An important aspect of QC’s action is its effect on non-specific immunity parameters. In groups QC1 and QC2, significant decreases in monocyte and neutrophil levels were observed. As shown in studies by several authors [60, 61], these changes are linked to QC’s ability to inhibit the synthesis of pro-inflammatory cytokines (IL-1β, TNF-α) by 40%–45% and to stimulate IL-10 production by 25%–30%.

Notably, our assessment of inflammatory status was based on hematological parameters. Although these are reliable indicators of systemic immune activation, future studies including specific cytokine assays (e.g., IL-6, TNF-α) would offer a more detailed understanding of the molecular mechanisms behind the immunomodulatory effects of QC.

The behavior of eosinophils is particularly noteworthy; their levels decreased in QC1 compared to the control. This change could have important practical implications. According to Deng *et al*. [62], a reduction in eosinophil levels is linked to a 15%–20% decrease in the frequency of allergic reactions and respiratory diseases in poultry. Changes in red blood parameters under QC influence were less obvious but still meaningful. In group QC1, hemoglobin concentration increased by 12.6% (121.25 g/L vs. 107.67 g/L in the control), and hematocrit rose by 4.1% (28.43% vs. 24.33%). These findings align with Wang *et al*. [63], who suggest that this effect results from QC’s ability to boost iron absorption in the intestine by 20%–25% and stimulate erythropoietin production by 15%–18%.

### Regional context and study limitations

The regional context of this study, carried out in the Southern Urals of Russia, is an important factor to consider. The BD used in our trial was based on locally predominant ingredients (such as specific types of wheat and soybean meal), which may have different nutritional and non-starch polysaccharide profiles compared to feed bases used in other regions. These compositional differences could significantly influence the structure and function of the gut microbiota. Therefore, the optimal QC dose identified here (5 mg/kg) may reflect a specific interaction with the local feed base and its associated microbial ecosystem.

This highlights the importance of conducting region-specific trials to tailor phytobiotic supplementation strategies, as a universal approach may not account for these vital agroecological and nutritional factors. Our findings offer useful insights for poultry production systems that utilize similar feed resources in comparable climatic zones. While this study examines the dose-dependent effects of QC on the gut–microbiome–immune axis in broilers, it also opens several promising avenues for future research. To expand on these results, future studies could investigate the synergistic effects of combining QC with probiotics or essential oils to improve its eubiotic and pathogen-suppressing properties.

Furthermore, comprehensive assessments of long-term carcass yields and meat quality are essential to establish a direct link between microbiome changes and concrete food safety and product quality outcomes, such as reduced pathogen levels and extended shelf life. Lastly, for mechanistic validation of the suspected microbiota–immune system interaction, targeted analysis of the expression of key immune pathway genes and cytokines in the gut and spleen is necessary. This would provide a deeper understanding of the molecular mechanisms behind the immunomodulatory effects of QC.

Although this study provides strong evidence for QC’s effectiveness, several limitations should be noted: the relatively short trial duration, the use of a single broiler breed, and the lack of direct biochemical markers of inflammation.

### Alpha- and beta-diversity analyses

An interesting aspect of our study is the lack of a significant effect of QC on alpha-diversity of the microbiome. Despite notable dose-dependent shifts in taxonomic structure at the phylum and genus levels, the phylogenetic richness and diversity indices remained statistically unchanged. This indicates that QC does not cause overall depletion or enrichment of the microbial community but instead exerts targeted, selective modulation, altering the ratio of already existing taxa.

A redistribution effect without a significant loss of overall diversity is considered favorable because high alpha-diversity is typically linked to gut ecosystem stability and resilience [64]. The notable changes in beta-diversity clearly show that QC has a targeted impact on microbiota composition, encouraging the development of unique microbial profiles in the experimental groups compared to the control. This supports the main conclusion that the cecal microbiome is dose-dependently modulated, with significant restructuring of the bacterial community even in the absence of alterations in overall diversity.

### Practical significance and future prospects

The results obtained have important implications for modern poultry production. Using QC at a dose of 5 mg/kg of feed not only enhances production indicators but also reduces the risk of metabolic disorders, particularly in intensive farming conditions. Studies have shown that dietary flavonoid supplementation can reduce the incidence of ascites and fatty liver syndrome [65].

The potential of QC as an alternative to antibiotic growth promoters is particularly noteworthy. Based on available data, flavonoid supplementation can reduce prophylactic antibiotic doses without harming productivity or survival [66]. This supports the current trend of decreasing antibiotic use in animal husbandry.

Therefore, the results of this study and the literature indicate that adding plant-derived QC to broiler diets is a promising strategy for antibiotic-free poultry production. It helps reduce mortality, improves feed efficiency, and increases daily live weight gain by positively affecting the gut microbiome. Although a large amount of data has been gathered, several issues still need more research. Future studies should explore the combined use of QC with other bioactive substances, refine administration methods across different growth stages, develop cost-effective methods to incorporate it into feed, and assess its long-term effects on product quality.

Furthermore, future studies should include cytokine profiling, quantitative assessment of short-chain fatty acids (SCFA), and long-term evaluations of meat quality to verify the microbiome-related benefits of QC.

## CONCLUSION

This study clearly shows a strong dose-dependent effect of dietary quercetin (QC) supplementation on broiler chicken performance, immune status, and cecal microbiota composition. Supplementing with QC at 5 mg/kg of feed yielded the most favorable results, including significantly higher final body weight, improved ADG, better FCR, and the highest EPEF compared to the control group. Hematological analysis indicated beneficial immunomodulatory effects, evidenced by increased total leukocyte and lymphocyte counts, along with reduced neutrophil, monocyte, and eosinophil levels, suggesting decreased systemic inflammatory responses. Microbiome profiling demonstrated targeted modulation of the cecal bacterial community, with enrichment of beneficial butyrate-producing genera such as *Faecalibacterium* and maintenance of overall microbial diversity, while higher QC doses (10–15 mg/kg) led to an increase in potentially pathogenic *Campylobacterota*.

From a practical standpoint, these findings identify 5 mg/kg of feed as an optimal and safe dietary level for QC in AA broilers. At this dose, QC improves growth performance, feed efficiency, livability, and immune balance without causing microbial imbalance. The observed gains in production efficiency and reductions in inflammatory markers are especially important for intensive poultry systems, where small improvements in feed conversion and survival yield significant economic benefits. Importantly, targeted modulation of the microbiome supports the use of QC as a plant-based alternative to antibiotic growth promoters, aiding antimicrobial stewardship and sustainable poultry farming.

A key strength of this study is its integrated experimental design, which combines growth performance metrics, detailed hematological profiling, and high-resolution *16S rRNA* gene sequencing of the cecal microbiome within a single framework. This comprehensive approach enables the identification of mechanistic links along the gut–microbiome–immune–performance axis and clarifies a clear dose–response relationship. The use of highly purified QC dihydrate further strengthens the conclusions by reducing variability in plant extract composition and bioavailability.

Despite these strengths, several limitations need to be recognized. The study was conducted over a relatively short production cycle and involved a single broiler genotype under controlled conditions, which may limit the generalizability of the results to other breeds or commercial settings. Additionally, immune and inflammatory status was mainly inferred from hematological parameters, without directly measuring cytokines, SCFA, or oxidative stress biomarkers. These factors restrict a more detailed mechanistic understanding at the molecular level.

Future research should concentrate on validating these findings under commercial production settings and across various broiler genotypes and feeding systems. Studies combining QC with other phytogenic compounds or probiotics may further improve its eubiotic and pathogen-inhibiting effects. Long-term evaluations of carcass traits, meat quality, and food safety outcomes are also necessary. Additionally, targeted analyses of cytokine expression, QS pathways, and short-chain fatty acid production would offer a deeper understanding of the molecular mechanisms behind QC’s immunomodulatory and microbiome-influenced effects.

In conclusion, this study provides strong evidence that dietary QC, when administered at the appropriate dose, can enhance growth performance, modulate immune responses, and positively shape the cecal microbiome of broiler chickens. By identifying a clear phytobiotic optimal level and emphasizing the dangers of excessive supplementation, these findings provide a scientifically sound basis for the rational use of QC as part of antibiotic-free poultry production strategies aligned with One Health principles.

## DATA AVAILABILITY

Raw sequencing data can be requested via email at icis-ofrc@list.ru, which belongs to the Institute for Cellular and Intracellular Symbiosis of the Ural Branch of the Russian Academy of Sciences (Orenburg, Russia).

## AUTHORS’ CONTRIBUTIONS

SR and MK: Conducted the experiments, analyzed and interpreted the data, and drafted the manuscript. DK: Performed mathematical processing of experimental data. DD and GD: Managed overall supervision and development of experimental methods. All authors have read and approved the final version of the manuscript.
